# Advancing Epidermal Barrier Resilience in Atopic Dermatitis with Isosorbide Di-Fatty Acid Esters: From Disruption to Restoration

**DOI:** 10.3390/biom16091246

**Published:** 2026-08-27

**Authors:** Ratan K. Chaudhuri, Raja K. Sivamani

**Affiliations:** 1 CuralysMD Dermaceuticals, A Hallstar Company, 10 Waterview Blvd, Parsippany, NJ 07054, USA; 2 Integrative Skin Science and Research, Sacramento, CA 95815, USA; raja.sivamani.md@gmail.com; 3 Integrative Research Institute, 4825 J Street, Suite 100, Sacramento, CA 95819, USA; 4 College of Medicine, California Northstate University, Elk Grove, CA 95757, USA; 5 Pacific Skin Institute, Sacramento, CA 95815, USA

**Keywords:** atopic dermatitis, epidermal barrier, skin resilience, isosorbide fatty acid diesters, microbiome, pruritus signaling

## Abstract

Atopic dermatitis (AD) is a chronic, relapsing inflammatory skin disease characterized by epidermal barrier dysfunction, immune dysregulation, microbial imbalance, and severe pruritus. Emerging evidence establishes that barrier disruption is a central pathogenic driver capable of initiating inflammatory signaling, neuroimmune activation, and chronic disease instability. This understanding has shifted therapeutic paradigms toward barrier-directed strategies aimed at restoring epidermal resilience. This narrative review evaluates the mechanistic and clinical evidence surrounding isosorbide fatty acid diester molecules—specifically isosorbide dicaprylate (IDC) and isosorbide di-(linoleate/oleate) (IDL)—as a barrier-first approach for AD management. Early in vitro and ex vivo investigations demonstrated that IDC significantly improves epidermal hydration, transepidermal water loss, and the expression of barrier-associated genes linked to epidermal integrity. Subsequent studies showed that IDL expands these effects through coordinated regulation of keratinocyte differentiation, lipid homeostasis, and inflammatory stress pathways. Furthermore, recent mechanistic data highlight synergistic anti-inflammatory and pruritus-modulating effects involving TRPA1-, TRPV3-, and TSLP-associated pathways, while preserving tissue integrity under cytokine-induced stress. Clinically, these findings are supported by randomized studies in pediatric and adult cohorts demonstrating significant reductions in pruritus, favorable Eczema Area and Severity Index (EASI) responses, decreased topical corticosteroid dependence, and a reduction in the relative abundance of *Staphylococcus aureus*. Collectively, these findings support a barrier-first therapeutic framework in which restoration of epidermal resilience may beneficially influence multiple interconnected pathways involved in atopic dermatitis.

## 1. Introduction

Atopic dermatitis (AD) is a chronic, relapsing inflammatory skin disorder characterized by epidermal barrier dysfunction, immune dysregulation, and recurrent cycles of disease flare and remission [1]. Globally, AD affects up to 20% of children and 3% to 5% of adults [2,3]. Increasing evidence suggests that AD extends beyond a localized skin condition, representing a systemic inflammatory process driven by interconnected epithelial, immunologic, microbial, and neurocutaneous pathways [4]. Traditionally, therapeutic paradigms focused reactively on suppressing inflammation after flare initiation. However, growing evidence indicates that epidermal barrier disruption is a central pathogenic driver—rather than a secondary consequence—that actively amplifies immune activation, microbial dysbiosis, and neurogenic pruritus [5,6]. Consequently, the therapeutic landscape is shifting from reactive immunosuppression toward proactive, long-term disease management focused on restoring epidermal integrity.

The epidermal barrier represents a highly dynamic biological system responsible for regulating hydration, keratinocyte differentiation, lipid organization, microbial homeostasis, and immune surveillance, while preventing transepidermal water loss (TEWL) [7]. Disruption of these functions elevates TEWL, reduces stratum corneum hydration, alters skin surface pH, and permits the penetration of environmental allergens, culminating in progressive barrier instability. Consequently, atopic dermatitis is increasingly recognized as a chronic state of impaired epidermal resilience driven by this interconnected barrier, inflammatory, and neurocutaneous dysfunction [8].

To address this multifaceted dysfunction, research over the past decade has focused on standardized isosorbide fatty acid diesters, transitioning from initial studies on hydration recovery toward broader insights into cellular differentiation and inflammatory modulation. Early evidence demonstrated that isosorbide dicaprylate (IDC) enhances skin hydration and upregulates barrier-related genes, including pathways associated with aquaporin-3 (AQP3), CD44, epidermal differentiation, and lipid metabolism [9]. Subsequent investigations revealed that isosorbide di-(linoleate/oleate) (IDL) further promotes the expression of key structural proteins, including filaggrin (FLG) and involucrin (IVL), while simultaneously suppressing interferon-associated signaling pathways linked to disrupted epidermal homeostasis [10]. Importantly, the distinct biological activities observed with isosorbide fatty acid diesters relative to structurally related fatty acid esters suggest that the molecular scaffold itself contributes to biological function, highlighting the importance of structure–function relationships in the development of barrier-directed skin therapeutics.

Further mechanistic studies utilizing cytokine-induced tissue models of atopic dermatitis demonstrated that IDC and IDL exert complementary and synergistic effects on epidermal integrity, inflammatory signaling, and neurogenic pruritus pathways. Specifically, these molecules modulate tumor necrosis factor-alpha (TNF-α)-responsive mediators and transient receptor potential ankyrin 1 (TRPA1)-associated itch signaling [11]. These mechanistic observations were subsequently validated by randomized, double-blind clinical trials in both adult and pediatric populations. In these clinical studies, topical formulations containing IDC and IDL significantly alleviated pruritus, reduced topical corticosteroid dependence, decreased the relative abundance of *Staphylococcus aureus*, and lowered overall disease severity while improving patient quality of life [12,13].

These findings support an evolving therapeutic perspective in which the restoration of epidermal barrier integrity actively influences multiple interconnected pathways governing atopic dermatitis pathophysiology. Building on the established “outside–inside” paradigm [14], this review advances a comprehensive barrier-resilience framework. Within this model, epidermal barrier dysfunction operates not merely because of inflammation, but as a central amplifying interface linking hydration failure, impaired keratinocyte differentiation, microbial dysbiosis, and neuroimmune pruritus signaling. Over the past decade, mechanistic, translational, and clinical investigations have progressively explored whether targeted structural reinforcement of the epidermal barrier can alter the biological microenvironment that drives chronic skin inflammation [15]. By shifting focus from reactive post-flare immunosuppression to proactive barrier stabilization, these studies have evaluated the capacity of barrier-directed interventions to optimize hydration, normalize differentiation pathways, and mitigate inflammatory cascades. This narrative review synthesizes a decade of scientific evidence on standardized isosorbide fatty acid diesters, positioning barrier-directed resilience biology as an integrated, evidence-based strategy for long-term disease management.

This review is not intended to provide a comprehensive overview of all biological mechanisms underlying epidermal barrier dysfunction in atopic dermatitis. Rather, it focuses on the mechanistic, preclinical, and clinical evidence supporting the role of the isosorbide di-fatty acid esters—isosorbide dicaprylate (IDC) and isosorbide dilinoleate/oleate (IDL)—in restoring epidermal barrier resilience, while providing only the biological background necessary to place these findings in context.

## 2. Methods

A comprehensive literature search was conducted in January 2026 using PubMed/MEDLINE and Google Scholar, supplemented by manual review of reference lists from relevant publications. The search targeted peer-reviewed, English-language mechanistic, translational, and clinical studies published from database inception through December 2025. Searches combined disease- and barrier-related terms with intervention-specific terms using Boolean operators, including (“atopic dermatitis” OR “epidermal barrier” OR “skin barrier” OR “keratinocyte differentiation” OR “skin microbiome” OR “pruritus”) AND (“isosorbide fatty acid diesters” OR “isosorbide dicaprylate” OR “IDC” OR “isosorbide dilinoleate/oleate” OR “IDL”). Additional searches combined intervention-specific terms with “hydration,” “transepidermal water loss,” “inflammation,” “TRPA1,” “TRPV3,” “TSLP,” “*Staphylococcus aureus*,” and “skin resilience.”

**Figure 1:**
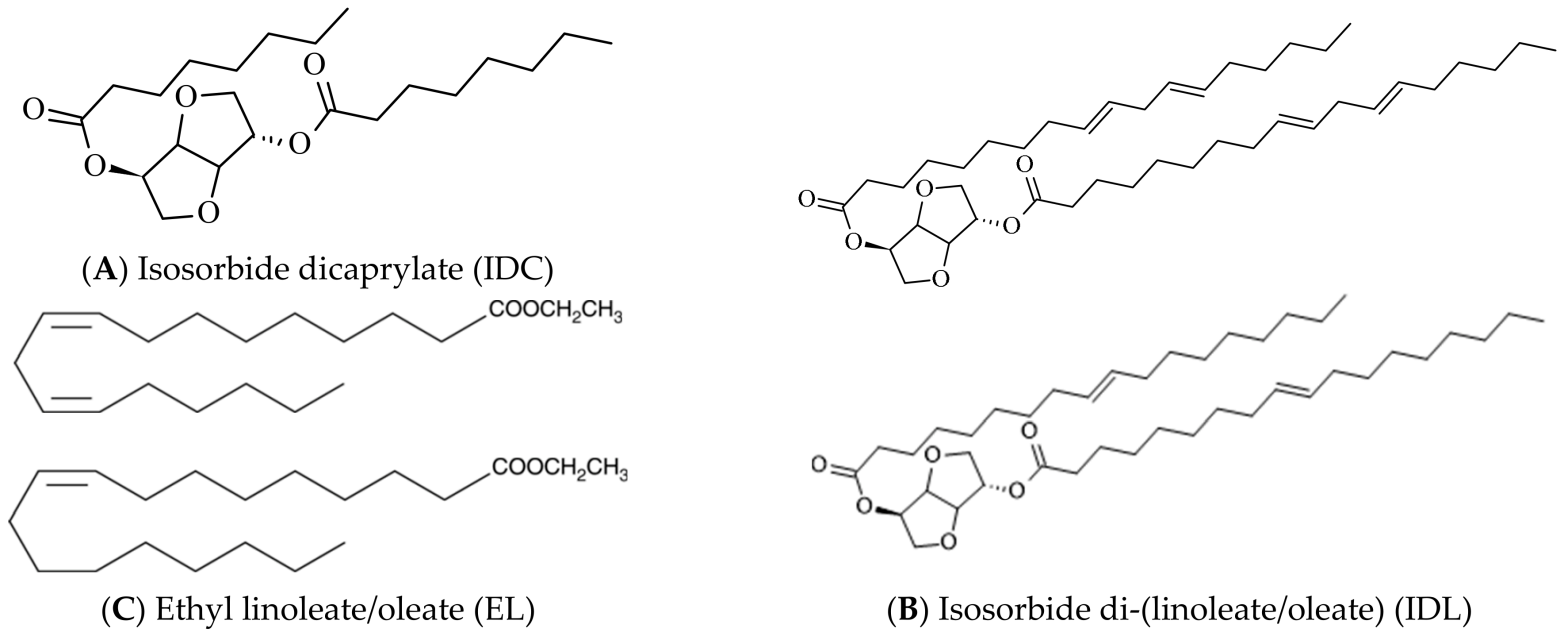
Chemical structures of isosorbide dicaprylate (IDC), isosorbide di-(linoleate/oleate) (IDL), and ethyl linoleate/oleate (EL). The common isosorbide scaffold present in IDC and IDL distinguishes these molecules from structurally simpler fatty acid esters such as EL. Differences in biological activity among these molecules suggest that the isosorbide scaffold contributes to biological function beyond fatty acid composition alone.

Studies were selected based on their relevance to epidermal barrier biology in atopic dermatitis and to the mechanistic, preclinical, translational, or clinical evaluation of IDC and IDL (Figure 1). Publications lacking sufficient methodological detail, non-peer-reviewed reports, conference abstracts, duplicate publications, and studies not directly relevant to the objectives of this review were excluded. The final evidence base included six core publications directly evaluating IDC and/or IDL, encompassing mechanistic, translational, and clinical investigations. An additional 45 publications were cited to provide mechanistic and biological background relevant to epidermal barrier function, inflammation, microbiome biology, pruritus, and atopic dermatitis, resulting in 51 references overall. Because this article is a focused narrative review rather than a systematic review, publications were selected for their scientific relevance and contribution to the mechanistic framework presented rather than through a formal systematic screening or quantitative evidence-synthesis process.

## 3. Atopic Dermatitis Beyond the Skin Barrier: Systemic and Neurocutaneous Interfaces

Increasing evidence suggests that AD extends beyond a disorder confined solely to the skin and instead represents a broader systemic inflammatory condition linked to chronic epidermal barrier dysfunction and immune dysregulation. Epidemiologic studies have long demonstrated associations between AD and other atopic disorders, including allergic rhinitis, asthma, and food allergy—a sequence described as the “atopic march” [4]. More recently, AD has been linked to a broader spectrum of systemic comorbidities, including cardiovascular disease, sleep disturbances, chronic inflammatory metabolic conditions, and alterations in systemic immune signaling pathways [16]. These observations support the concept that persistent epidermal barrier disruption does not merely drive localized cutaneous inflammation but can contribute to broader systemic inflammatory amplification through chronic neuroimmune signaling, microbial dysbiosis, oxidative stress, and sustained cytokine release.

Consistent with this perspective, several circulating biomarkers associated with T-helper 2 (Th2)-driven inflammation correlate with disease severity, pruritus intensity, and therapeutic response in AD. These include eosinophil-associated mediators and chemokine (C-C motif) ligand 17 (CCL17), also known as thymus and activation-regulated chemokine (TARC) [17]. In parallel, increasing recognition of the complex interplay among immune signaling, microbiome alterations, oxidative stress, keratinocyte stress responses, and neurocutaneous pathways has expanded the understanding of AD from localized inflammatory dermatosis toward a multifactorial disorder. Furthermore, numerous intracellular regulatory mechanisms, including microRNA-mediated pathways, contribute to AD pathogenesis, underscoring the disease as a dynamically integrated disorder involving systemic immune dysregulation and reciprocal interactions with epidermal keratinocytes [18].

Collectively, these observations support the emerging concept that restoration of epidermal barrier integrity may influence not only local inflammatory processes within the skin, but also broader biological pathways associated with chronic disease amplification. Within this evolving framework, it becomes critical to evaluate the mechanistic, translational, and clinical potential of topical barrier-directed strategies designed to restore skin homeostasis. Consequently, addressing the fundamental physiological deficits of the barrier represents the vital next step in long-term disease management.

## 4. Molecular Mechanisms Underlying IDC-Induced Hydration and Macromolecular Barrier Recovery

Initial research into this therapeutic paradigm focused on whether the modulation of epidermal water homeostasis could influence broader aspects of skin barrier integrity and function. Historically, skin hydration was often viewed primarily through a cosmetic lens, centered largely on transient moisturization and the symptomatic relief of dryness. However, substantial evidence has established that stratum corneum hydration plays a fundamental biological role in maintaining epidermal permeability barrier function, keratinocyte differentiation, lipid organization, enzymatic activity, and overall tissue resilience [19,20]. Studies have shown that increased skin hydration improves wound repair and healing [21].

Within this context, IDC—a novel diester derived from isosorbide and caprylic acid—was investigated for its specific effects on hydration-associated pathways and epidermal barrier biology [9]. Microarray gene expression profiling revealed a coordinated upregulation of aquaporin-3 (AQP3), cluster of differentiation 44 (CD44), E-cadherin (CDH1), ceramide synthase 3 (CERS3), small proline-rich region protein 3 (SPRR3), and multiple late cornified envelope (LCE) proteins, including LCE1E, LCE3A, LCE3B, LCE3C, and LCE3D. While AQP3 and CD44 are closely associated with epidermal hydration and homeostasis [22], CDH1 and CERS3 contribute critically to epithelial cohesion and lipid barrier organization, respectively [23,24,25]. Beyond its role in maintaining epidermal hydration, CD44 serves as an important regulator of epidermal differentiation, lipid homeostasis, and barrier repair through hyaluronic acid (HA)-dependent signaling pathways. This suggests that IDC-mediated upregulation of CD44 may have broader implications for epidermal resilience than initially appreciated [26,27].

In parallel, SPRR3 and LCE proteins represent vital components of the cornified envelope involved in terminal keratinocyte differentiation, adaptive barrier repair responses, and structural reinforcement of the epidermal barrier [28]. Particularly noteworthy was the induction of LCE3B and LCE3C, members of an epidermal stress-response gene cluster implicated in barrier repair and inflammatory disorders [29]. Several of these key observations, including the upregulation of LCE1E, LCE3D, CERS3, SPRR3, and CDH1, were independently validated by quantitative polymerase chain reaction (qPCR), while increased AQP3 expression was confirmed at the protein level [9]. These findings indicated that IDC influences multiple interconnected pathways governing hydration, epidermal maturation, lipid organization, and barrier resilience rather than functioning solely as a conventional moisturizing agent.

These mechanistic findings are strongly supported by clinical data. Topical application of IDC-containing formulations improved skin hydration and significantly reduced TEWL, indicating a true enhancement of epidermal permeability barrier function rather than transient surface hydration alone [9]. In comparative studies, IDC demonstrated superior and prolonged hydration effects relative to glycerol-containing control formulations, with clinical benefits persisting beyond the active treatment period.

In summary, these findings highlight an important physiological principle: the restoration of hydration and barrier homeostasis influences multiple interconnected biological processes governing epidermal stability. Rather than functioning solely as a passive emollient, IDC modulates pathways associated with barrier organization, differentiation, and tissue homeostasis. These findings provided an important conceptual foundation for subsequent investigations into inflammation, pruritus signaling, and chronic barrier dysfunction in atopic dermatitis.

## 5. IDL-Mediated Restoration of Epidermal Barrier Function

Although IDC demonstrated important effects on hydration-associated pathways and epidermal barrier recovery by stabilizing tight junctions and modulating aquaporin expression, subsequent molecular investigations suggested that the comprehensive restoration of long-term barrier resilience likely required a broader, more systemic regulation of keratinocyte terminal differentiation and stratum corneum macromolecular organization [30]. This technical realization led to the engineering and development of IDL, generated through the targeted esterification of a core isosorbide scaffold with sunflower-derived fatty acids consisting of approximately 65% linoleic acid and 20% oleic acid. Importantly, these comparative structural studies demonstrated that not all linoleate/oleate esters exert equivalent biological or phenotypic effects on epidermal homeostasis, revealing that the molecular backbone carries critical functional significance [10].

Comparative transcriptomic profiling and DNA microarray analyses revealed that IDL produced substantially different, highly prioritized biological responses than ethyl linoleate/oleate (EL), despite their nearly identical fatty acid inputs [10]. Whereas ethyl linoleate/oleate primarily influenced localized lipid metabolic pathways and targeted inflammatory signaling cascades, IDL demonstrated a superior ability to activate highly coordinated gene networks and co-expression modules linked directly to architectural epidermal differentiation, advanced keratinization, cell–cell desmosomal adhesion, and comprehensive stratum corneum structural organization.

Interestingly, micro-array and RT-PCR verification confirmed that IDL upregulated multiple differentiation-associated genes essential to macro-structural skin resilience. These included FLG, involucrin IVL, LOR, aquaporin 9 (AQP9), keratin-1 (KRT1), and the core transcription factor grainyhead-like 2 (GRHL2) [10]. Concurrently, IDL strongly stimulated the small proline-rich region (SPRR) multigene family embedded within the EDC, showing efficacy toward SPRR3 and SPRR4 [10]. These synchronized gene targets may contribute directly to cornified envelope (CE) macromolecular formation, keratinocyte terminal differentiation, adaptive epidermal stress responses, and the structural integrity of the outer skin barrier [10]. Furthermore, treatment with IDL was shown to effectively downregulate a distinct “unhealthy skin signature (USS)” gene module while uniquely repressing interferon (IFN)-inducible networks that disrupt epidermal balance during chronic stress. On an ultrastructural level, this dual mechanistic action—the simultaneous upregulation of structural envelope proteins and the suppression of barrier-degrading cytokine signaling—prevents the breakdown of the stratum corneum matrix, thereby maintaining optimal desmosomal linkage and preventing premature corneocyte desquamation.

The biological significance of SPRR4 may extend far beyond its established, canonical role as a small proline-rich cell-cortex envelope protein that serves as a substrate for transglutaminase cross-linking. It contributes uniquely to the mechanical reinforcement of the cornified envelope and offers active cellular protection against environmental oxidative stress through inherent, highly active antioxidant properties [31]. Strikingly, evolutionary genomic analyses of the epidermal differentiation complex (EDC) have highlighted the evolutionary pressure on this locus, identifying SPRR4 as one of the most consistently and strongly positively selected barrier-related genes across mammalian lineages [32]. Importantly, IDL not only increased expression of differentiation-associated genes but also protected against cytokine-mediated stratum corneum degradation [33]. In parallel, IDL suppressed USS genes [34] and downregulated interferon-associated inflammatory modules linked to disruption of epidermal homeostasis implicated in chronic inflammatory stress responses [35]. These observations further supported the concept that IDL was influencing coordinated inflammatory and barrier-regulatory pathways rather than functioning solely as a conventional emollient. These findings broadened the conceptual framework of barrier-directed therapy beyond hydration alone toward coordinated regulation of epidermal differentiation, structural barrier organization, inflammatory modulation, and tissue resilience.

In explant studies using human epidermal tissue, topical application of 4% IDL increased epidermal abundance of AQP9, FLG, and LOR by 94%, 13%, and 12%, respectively [10]. Equally important, IDL preserved stratum corneum integrity under inflammatory stress conditions and prevented cytokine-mediated barrier degradation while suppressing inflammatory signaling pathways associated with T-cell activation, adaptive immunity, and NF-κB activation. In cytokine-stimulated epidermal tissue models, IDL preserved stratum corneum integrity and prevented cytokine-mediated barrier degradation, consistent with a barrier-protective effect also observed with retinoic acid as a positive control. These observations suggest that restoration of epidermal homeostasis requires not only enhancement of differentiation-associated pathways but also preservation of structural barrier integrity under chronic inflammatory conditions characteristic of atopic dermatitis.

Beyond keratinocyte differentiation and cornified envelope formation, IDL also influenced pathways involved in epidermal lipid homeostasis, another essential determinant of barrier integrity. Because effective barrier function depends not only on structural protein organization but also on coordinated lipid processing and ceramide production [14], targeted RT-PCR analyses further demonstrated significant upregulation of multiple genes involved in epidermal lipid metabolism and transport, including ATP-binding cassette transporter G1 (ABCG1), α/β-hydrolase domain-containing protein 5 (ABHD5), ceramide synthase 3 (CERS3), elongation of very long-chain fatty acids protein 2 (ELOVL2), and low-density lipoprotein receptor adaptor protein 1 (LDLRAP1). Importantly, ABHD5 has recently been proposed to function as a spatial organizer of epidermal lipid synthesis by coordinating lipid droplet-derived substrates with PNPLA1, a patatin-like phospholipase essential for acylceramide biosynthesis, thereby facilitating the lipid-processing machinery required for epidermal barrier formation [36]. These findings indicate that IDL promotes coordinated regulation of both protein and lipid components of the epidermal barrier, supporting an integrated model of barrier restoration that encompasses both structural architecture and functional integrity.

Interestingly, these mechanistic and structural observations translated into measurable physiological improvements in human subjects. In a placebo-controlled, double-blinded clinical study involving subjects with xerotic skin, short-term treatment with a 2% IDL lotion significantly improved skin hydration within 1–2 weeks while simultaneously reducing TEWL, indicating enhanced epidermal permeability barrier function [10]. Importantly, improvements in hydration persisted beyond the active treatment period, suggesting that the observed effects extended beyond transient surface occlusion and may instead reflect restoration of underlying barrier homeostasis. Collectively, these findings suggest that coordinated regulation of keratinocyte differentiation, lipid homeostasis, structural proteins, and inflammatory stress responses can translate into measurable improvements in barrier resilience.

## 6. Integrated Biological Effects of IDC and IDL in Epidermal Restoration in an AD-like Tissue Model

While early findings focused on epidermal hydration and barrier structure, later studies showed that chronic barrier disruption in atopic dermatitis is closely linked to inflammatory amplification and neuroimmune pathways involved in tissue injury and itch. These insights led to investigation of whether isosorbide diesters could modulate inflammatory and pruritus-related pathways under AD-like conditions. In 2022, studies using cytokine-stimulated reconstructed human epidermis and ex vivo skin explant models with IL-4, IL-13, TNF-α, and IL-31 showed that IDL and IDC preserved epidermal architecture while reducing inflammatory responses associated with atopic dermatitis-like barrier dysfunction [11]. In this cytokine-induced AD-like tissue model, exposure to IL-4, IL-13, TNF-α, and IL-31 disrupted keratinocyte differentiation, produced a fissured epidermal architecture, and generated gene expression changes that resembled the atopic dermatitis transcriptome. Cumulatively, these findings established a biologically relevant AD-like platform for evaluating how IDL and IDC could preserve tissue integrity while modulating inflammatory and neuroimmune pathways associated with chronic barrier disruption.

Transcriptomic analyses demonstrated suppression of TNF-associated inflammatory signaling, with coordinated downregulation of mediators including IL1B and ITGA5, together with broader repression of interferon-responsive modules associated with epidermal stress [11]. Importantly, this selective barrier-restorative transcriptional profile differs from the broad glucocorticoid receptor-mediated transcriptional program characteristic of topical corticosteroids [37]. Unlike the broad T-cell-directed immunosuppressive effects of calcineurin inhibitors [38], the combined IDC + IDL response appears to selectively attenuate barrier injury-associated inflammatory networks while preserving the biological processes required for epidermal barrier restoration, consistent with a resilience-based rather than immunosuppressive mode of action.

Pruritus-related signaling provided an additional mechanistic dimension to these findings. TRPA1 and TRPV3 represent complementary neuroimmune and epidermal signaling nodes linking barrier dysfunction to chronic itch and inflammatory amplification [39,40]. The marked downregulation of TRPA1, especially with the IDL + IDC combination, accompanied by modulation of TRPV3-associated pathways, suggests potential interruption of itch–scratch cycle biology by dampening signals that reinforce neuroimmune activation, barrier injury, and secondary inflammatory escalation. In this way, modulation of pruritus signaling may have extended beyond symptomatic itch control to influence the broader network of epidermal stress responses that perpetuate chronic atopic dermatitis.

A further layer of evidence supporting tissue resilience came from markers of cellular stress and injury. In ex vivo inflammatory challenge models, IDC was associated with a greater than 50% reduction in measured LDH release [11,41]. However, because both IDL and IDC also directly inhibited LDH enzymatic activity in vitro, this reduction cannot be interpreted as independent evidence of decreased cellular injury and should therefore be considered cautiously. In the context of the accompanying histological and molecular findings, the LDH observation is nevertheless consistent with the broader evidence of preserved epidermal architecture under cytokine-induced stress. Clinically, elevated serum LDH has been associated with greater disease severity and poorer therapeutic outcomes in atopic dermatitis [42,43], supporting the biological relevance of LDH as a marker of tissue injury, while not altering the methodological limitation of the ex vivo LDH measurement. Effects on thymic stromal lymphopoietin (TSLP), a key epithelial-derived cytokine involved in inflammatory amplification and itch-associated signaling [39], were more modest but trended in the same protective direction, suggesting partial attenuation of epithelial stress signaling rather than broad immunosuppression. Interestingly, the Th2/TNF-α inflammatory model also demonstrated increased expression of mitochondrial inner membrane and respiratory chain-associated genes, recapitulating metabolic features previously reported in non-lesional AD skin and further supporting the biological relevance of the model [44]. Taken together, the histological and molecular findings support a broader role for isosorbide diesters in preserving epidermal integrity, attenuating maladaptive stress responses, and promoting barrier resilience under conditions that mimic chronic atopic dermatitis.

In summary, these studies demonstrate that epidermal barrier disruption, inflammatory amplification, neuroimmune activation, and pruritus are deeply interconnected components of atopic dermatitis pathophysiology. Rather than functioning solely as passive moisturization agents or broad immunosuppressive therapies, isosorbide fatty acid diesters appear to influence coordinated biological networks governing tissue integrity, inflammatory stress responses, keratinocyte injury, and itch-associated signaling pathways. These observations support an emerging systems-level framework in which restoration of epidermal resilience represents a unifying strategy to interrupt self-reinforcing cycles of barrier dysfunction, inflammation, neurocutaneous stress responses, and persistent disease activity in atopic dermatitis.

## 7. Clinical Translation of IDC and IDL: Efficacy and Steroid-Sparing Outcomes in Adults

The mechanistic observations seen in tissue models began translating into clinically meaningful outcomes in a 4-week prospective, randomized, double-blind, vehicle-controlled adult study of mild-to-moderate atopic dermatitis [12]. In this trial, adults received either a vehicle emollient containing 0.1% colloidal oatmeal alone or the same base formulation supplemented with standardized isosorbide diesters (4% IDC + 4% IDL). The primary goal was to determine whether this barrier-directed combination could improve itch and disease severity while reducing reliance on topical corticosteroids under real-world inflammatory conditions.

Clinical efficacy findings were directionally consistent with the earlier barrier and anti-inflammatory data. Participants treated with IDC + IDL achieved a 65.6% improvement in itch compared with 43.8% in the vehicle group, a statistically significant difference at 4 weeks. In parallel, 56.5% of participants in the IDC + IDL group achieved EASI 75 compared with 25% in the vehicle group, indicating a favorable trend toward greater clinical improvement in overall disease severity. Although changes in hydration and TEWL did not significantly differ between groups, the findings in the pilot study suggest that expanded studies in atopic dermatitis with increased power are warranted.

One of the most clinically relevant findings was the reduction in topical corticosteroid dependence. Relative corticosteroid use increased substantially in the vehicle group, whereas it declined in the IDC + IDL group during the early treatment period, with significant separation between groups at week 1 and a continued favorable trend at week 2. This steroid-sparing pattern is particularly important in adult atopic dermatitis, where long-term management often depends on balancing flare control with efforts to minimize chronic corticosteroid exposure. In this context, the adult study provided translational support for the concept that barrier-first intervention may reduce the need for downstream topical anti-inflammatory rescue therapy during active disease.

The microbiome findings added another translational layer to these results. Treatment with IDC + IDL was associated with a reduction in the relative abundance of *S. aureus* at week 4, whereas no such change was observed with vehicle alone. Given the established relationship between *S. aureus* overgrowth, barrier disruption, and flare activity in atopic dermatitis, this observation suggests that standardized isosorbide diesters may help shift the cutaneous environment away from one that favors microbial dysbiosis and recurrent inflammatory aggravation [45]. The observations in this study are consistent with previous reports indicating that stabilization of the epidermal barrier, restoration of the skin’s naturally acidic pH, and attenuation of underlying inflammation collectively promote a reduction in the relative abundance of *S. aureus* [46,47]. These findings are especially notable because they align with the broader mechanistic framework in which barrier restoration, inflammatory modulation, and reduction in the relative abundance of *S. aureus* are interconnected biological consequences of improved epidermal barrier function rather than therapeutically separate processes.

Taken together, the adult clinical study marked an important point of translational validation. The coordinated improvements in itch, the favorable EASI 75 trend, the early steroid-sparing effect, and the reduction in *S. aureus* abundance all support the view that the scientific observations first seen in mechanistic tissue models were beginning to translate into clinically meaningful outcomes. Rather than acting simply as an emollient add-on, the IDC + IDL formulation appeared to function within a broader barrier-first management framework in which restoration of epidermal resilience may influence symptoms, inflammatory burden, microbial balance, and treatment dependence simultaneously.

## 8. Pediatric Clinical Evaluation and Reduction in *Staphylococcus aureus*

These adult findings set the stage for an important next question: whether the same barrier-first benefits could be reproduced in pediatric atopic dermatitis, where disease burdens, epidermal vulnerability, microbiome instability, and long-term treatment dependence often begin early in life. In pediatric populations, where safety, tolerability, and minimization of chronic corticosteroid exposure are particularly important considerations, development of effective barrier-directed strategies may hold added important clinical significance for the ongoing management of atopic dermatitis and maintenance of epidermal resilience.

A subsequent 8-week randomized, double-blind, vehicle-controlled pediatric study evaluated the effects of standardized isosorbide diesters combined with colloidal oatmeal in children aged 2–17 years with mild-to-moderate atopic dermatitis [13]. Participants received either a colloidal oatmeal lotion alone or the same formulation supplemented with IDL and IDC, while topical hydrocortisone 2.5% was permitted as rescue therapy when needed. The study assessed disease severity, itch, sleep, topical corticosteroid usage, skin hydration, and microbiome changes over time.

Clinical outcomes in the pediatric population were directionally consistent with earlier mechanistic observations and adult clinical findings. Treatment with IDL + IDC was associated with statistically significant improvements in pruritus, including a significantly greater proportion of participants achieving a ≥4-point reduction in IVAS itch at both week 4 (45.5% vs. 6.3%; *p* = 0.0085) and week 8 (42.9% vs. 12.5%; *p* = 0.045), together with a significant improvement in Dermatology Life Quality Index (DLQI) at week 8 (*p* = 0.035). Improvements in EASI50 and EASI75 also favored the IDL + IDC group (81.0% vs. 56.3% and 42.9% vs. 18.8%, respectively) but did not reach statistical significance, likely reflecting the limited sample size of this exploratory study. These findings suggest that restoration of epidermal barrier function may translate into clinically meaningful reductions in itch burden during the treatment period.

One of the most clinically important observations was the marked reduction in topical corticosteroid use in the IDL + IDC group. Children receiving the barrier-directed formulation used substantially less cumulative topical corticosteroid throughout the study period compared with vehicle-treated participants, while a greater proportion of subjects avoided corticosteroid use entirely. These findings carry relevance in pediatric atopic dermatitis, where concerns regarding chronic corticosteroid exposure, skin sensitivity, tolerability, and long-term treatment burden frequently influence caregiver decision-making and therapeutic adherence.

The pediatric study also provided additional translational support for the emerging relationship among barrier integrity, microbiome dysbiosis, and inflammatory disease activity. Treatment with IDL + IDC reduced the relative abundance of *S. aureus* over the 8-week treatment period, whereas no comparable reduction was observed in the vehicle group. Given the established association between *S. aureus* overgrowth, epidermal barrier disruption, inflammatory amplification, and disease flare activity in atopic dermatitis [45], these observations further support the concept that restoration of epidermal resilience may help shift the cutaneous environment away from one that favors *S. aureus* overgrowth and recurrent inflammatory instability.

Notably, improvements observed in the pediatric study extended beyond investigator-assessed disease severity alone. Reduction in itch burden, improvement in sleep-associated measures, and trends toward improved quality-of-life outcomes highlight the broader clinical implications of barrier-directed management in children with atopic dermatitis. Because pediatric AD frequently affects not only the child but also caregiver stress, sleep quality, and family well-being, these findings further emphasize the importance of therapeutic approaches capable of supporting long-term disease control while minimizing treatment-related burden.

The pediatric findings provided important translational support for the evolving barrier-first hypothesis. The coordinated improvements in itch, disease severity, corticosteroid sparing, hydration, reduction in the relative abundance of *Staphylococcus aureus*, and quality-of-life outcomes collectively support the concept that restoration of epidermal resilience may beneficially influence multiple interconnected dimensions of atopic dermatitis biology. Rather than functioning solely as symptomatic moisturizers, standardized isosorbide diesters appear to operate within a broader framework of barrier-directed disease modulation by integrating improvements in epidermal integrity, inflammatory signaling, reduction in the relative abundance of *S. aureus*, and clinically meaningful patient outcomes.

## 9. Longitudinal Case Observation in Complex Atopic Dermatitis

Although randomized clinical studies provide important evidence regarding efficacy and mechanism, individual case observations can offer additional insight into how barrier-directed strategies perform under real-world conditions characterized by chronic flares, variable adherence, environmental triggers, and long-standing epidermal instability. In pediatric atopic dermatitis particularly, disease burden extends beyond visible skin manifestations alone and often significantly affects sleep quality, emotional well-being, caregiver stress, and overall family quality of life. Within this context, the following case study illustrates how restoration of epidermal resilience through a barrier-first approach may influence not only clinical symptoms, but also broader aspects of disease management in atopic dermatitis.

A published pediatric case report described a 9-year-old boy with moderate refractory atopic dermatitis who had persistent disease despite multiple prior topical therapies, including corticosteroids, tacrolimus, topical ruxolitinib, and topical tapinarof, together with recurrent secondary skin infections requiring oral antibiotics [48]. The family declined systemic therapy and initiated narrowband (NB) UVB phototherapy [49] with only partial improvement. Introduction of a standardized isosorbide diester lotion containing IDC, IDL, and colloidal oatmeal during ongoing NB-UVB treatment was associated with rapid clinical improvement within four weeks, with marked reduction in eczematous lesions and sustained improvement during continued daily use. Because of the concomitant NB-UVB therapy and the uncontrolled nature of this observation, this case should be regarded as hypothesis-generating rather than evidence of causality. Importantly, no additional prescription topical anti-inflammatory agents were required during the observation period.

Although the continued use of NB-UVB phototherapy prevents attribution of efficacy solely to the moisturizer intervention, the rapid improvement observed following introduction of the barrier-directed formulation aligns closely with earlier mechanistic, translational, and clinical observations described throughout this review. The case further illustrates how restoration of epidermal resilience may complement existing therapeutic strategies by supporting barrier integrity, reducing inflammatory stress amplification, and improving overall clinical management during ongoing treatment. Particularly in pediatric atopic dermatitis, where long-term safety, tolerability, and treatment burden remain major considerations for patients and caregivers alike, barrier-directed adjunctive approaches may provide clinically meaningful support within chronic disease management frameworks and overall clinical management during ongoing treatment.

In summary, this case study adds an additional translational dimension to the evolving barrier-centered hypothesis by illustrating how mechanistic insights involving hydration biology, keratinocyte differentiation, inflammatory modulation, microbiome balance, and tissue resilience may ultimately converge in real-world patient management. Rather than functioning solely as symptomatic moisturization, the observed clinical response further supports the concept that restoration of epidermal resilience may influence multiple interconnected aspects of atopic dermatitis biology simultaneously.

## 10. Paradigm Shift: From Reactive Inflammation Suppression to Proactive Barrier Resilience

Traditional therapeutic strategies in atopic dermatitis have focused predominantly on suppressing downstream inflammatory pathways after flare initiation. While these approaches remain clinically important, the findings summarized throughout this review support an expanded therapeutic perspective in which restoration of epidermal resilience itself may help reduce the biological conditions that perpetuate inflammatory amplification, microbial dysbiosis, neuroimmune activation, and chronic disease instability. Within this framework, the epidermal barrier is viewed not simply as a passive structural layer requiring symptomatic moisturization, but as an active biological interface capable of influencing multiple interconnected pathways governing cutaneous homeostasis.

These findings summarized in this review support an evolving therapeutic framework in which restoration of epidermal barrier integrity may influence multiple interconnected pathways governing atopic dermatitis pathophysiology, including hydration, keratinocyte differentiation, inflammatory amplification, microbiome balance, neuroimmune signaling, and tissue resilience. Over the past decade, the scientific understanding of isosorbide diesters has progressively evolved from simple observations of improved hydration and reduced TEWL toward a broader systems-level perspective integrating barrier biology, inflammatory regulation, and epidermal resilience.

**Figure 2:**
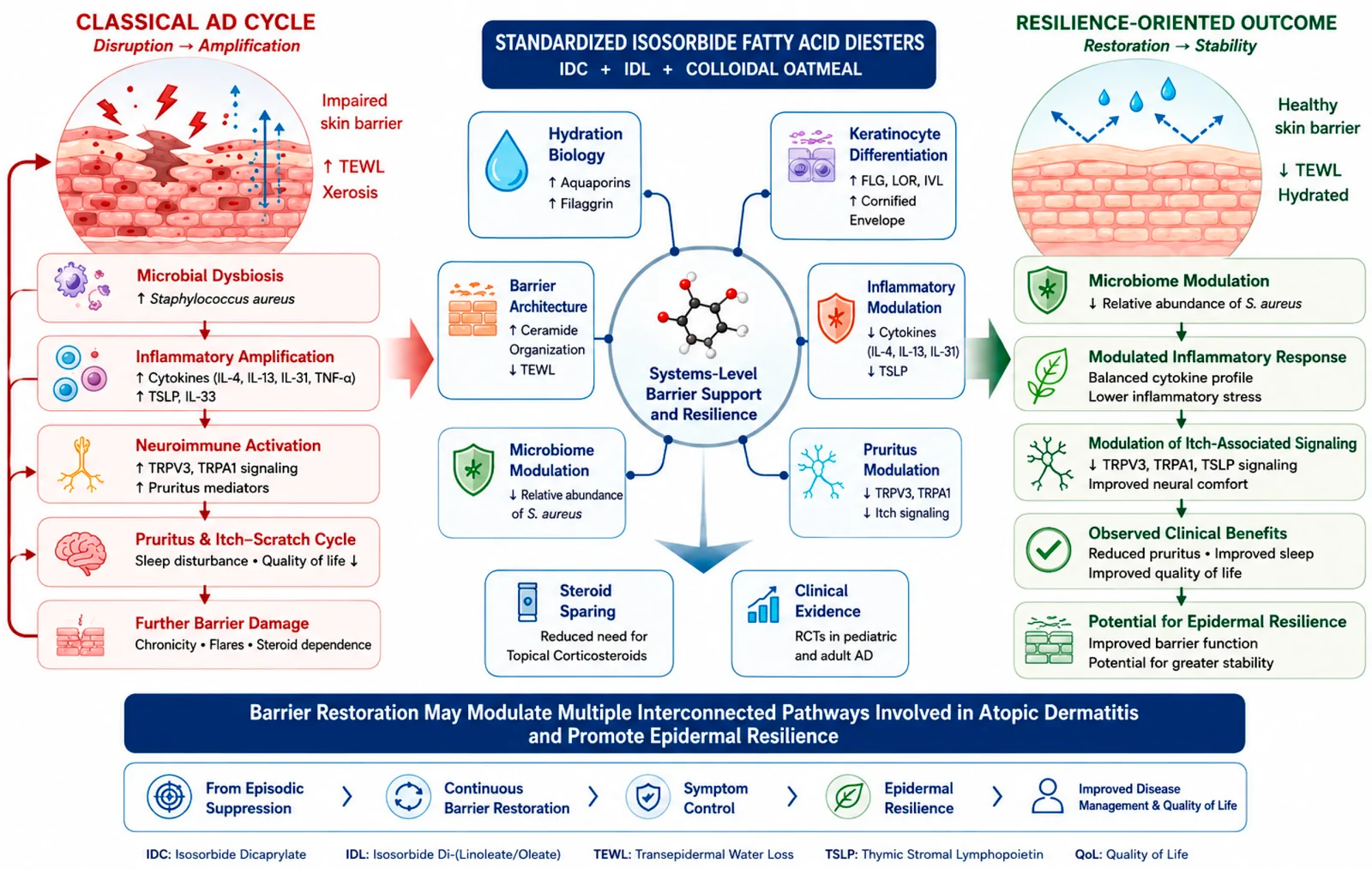
Potential barrier-first model for epidermal resilience in atopic dermatitis. Table proposed conceptual model illustrates how standardized isosorbide fatty acid diesters, including isosorbide dicaprylate (IDC) and isosorbide di-(linoleate/oleate) (IDL), may extend beyond hydration-focused barrier support by influencing interconnected pathways involved in epidermal differentiation, barrier architecture, inflammatory and pruritus signaling, and the relative abundance of *Staphylococcus aureus.* Based on the mechanistic, translational, and clinical evidence reviewed herein, these coordinated effects may support epidermal resilience and improved clinical management of atopic dermatitis.

In many ways, this work represents more than the development of topical formulations alone. It reflects a decade-long evolution of a barrier-centered scientific hypothesis that continues to expand through emerging mechanistic, translational, and clinical insights. Collectively, these findings support a paradigm in which restoration of epidermal resilience represents not merely a strategy for improving barrier function, but an upstream biological intervention that stabilizes the interconnected processes governing cutaneous homeostasis, inflammatory signaling, microbiome balance, neuroimmune communication, and long-term disease stability in atopic dermatitis (Figure 2).

## 11. Conclusions and Future Directions in Barrier-Directed Therapeutics

Over the past decade, our understanding of atopic dermatitis has evolved from a predominantly inflammation-centered model toward a more integrated view in which barrier disruption, inflammatory amplification, *S. aureus* overgrowth, and neurogenic itch signaling operate as interconnected processes. The development of a standardized isosorbide diester platform reflects this evolving perspective: beginning with investigations into hydration and epidermal barrier function and progressively expanding into keratinocyte differentiation, inflammatory modulation, reduction in the relative abundance of *S. aureus*, and clinical resilience. While further studies are warranted, emerging evidence suggests that barrier-directed strategies may play an increasingly important role in shifting eczema management from episodic suppression toward long-term restoration of skin resilience. Collectively, the available evidence supports the concept that restoration of barrier integrity may influence multiple biological processes central to eczema pathophysiology and provides a foundation for further investigation of epidermal resilience biology in chronic inflammatory skin disease.

The evolution of the isosorbide diester platform with colloidal oatmeal reflects an evolving understanding of atopic dermatitis as a disease characterized by intertwined inflammation and chronic barrier dysfunction, requiring continuous restoration of epidermal resilience. Rather than providing passive moisturization alone, barrier-directed therapies may help normalize the biological environment that drives itch amplification, microbial dysbiosis, inflammatory signaling, and recurrent disease flares. Future studies should determine whether earlier implementation of barrier-directed interventions can favorably influence the long-term clinical course of atopic dermatitis by reducing treatment escalation and improving disease control. Ultimately, the decade-long evolution of the isosorbide diester platform supports a broader conceptual shift in atopic dermatitis management—from treating the consequences of barrier dysfunction to restoring the biological resilience required to maintain long-term skin homeostasis.

## Data Availability

All data analyzed are from publicly available sources, as cited in the manuscript.
